# Genetic variation and recombination analysis of the *GP5* (*GP5a*) gene of PRRSV-2 strains in China from 1996 to 2022

**DOI:** 10.3389/fmicb.2023.1238766

**Published:** 2023-08-22

**Authors:** Qin Luo, Yajie Zheng, Yingxin He, Gan Li, Hang Zhang, Huiyang Sha, Zhiqing Zhang, Liangzong Huang, Mengmeng Zhao

**Affiliations:** School of Life Science and Engineering, Foshan University, Foshan, China

**Keywords:** PRRSV, *GP5* gene, *GP5a* gene, genetic variation, recombination, phylogeny

## Abstract

Porcine reproductive and respiratory syndrome virus (PRRSV) has been prevalent in China for more than 25 years and remains one of the most significant pathogens threatening the pig industry. The high rate of mutation and frequent recombination of PRRSV have exacerbated its prevalence, particularly with the emergence of highly pathogenic PRRSV (HP-PRRSV) has significantly increased the pathogenicity of PRRSV, posing a serious threat to the development of Chinese pig farming. To monitor the genetic variation of PRRSV-2 in China, the *GP5* sequences of 517 PRRSV-2 strains from 1996 to 2022 were analyzed and phylogenetic trees were constructed. Furthermore, a total of 60 PRRSV strains, originating from various lineages, were carefully chosen for nucleotide and amino acid homologies analysis. The results showed that the nucleotide homologies of the PRRSV *GP5* gene ranged from 81.4 to 100.0%, and the amino acid homologies ranged from 78.1 to 100.0%. Similarly, the PRRSV *GP5a* gene showed 78.0 ~ 100.0% nucleotide homologies and 70.2 ~ 100.0% amino acid homologies. Amino acid sequence comparisons of *GP5* and *GP5a* showed that some mutations, such as substitutions, deletions, and insertions, were found in several amino acid sites in *GP5*, these mutations were primarily found in the signal peptide region, two highly variable regions (HVRs), and near two T-cell antigenic sites, while the mutation sites of *GP5a* were mainly concentrated in the transmembrane and intramembrane regions. Phylogenetic analysis showed that the prevalent PRRSV-2 strains in China were divided into lineages 1, 3, 5, and 8. Among these, strains from lineage 8 and lineage 1 are currently the main prevalent strains, lineage 5 and lineage 8 have a closer genetic distance. Recombination analysis revealed that one recombination event occurred in 517 PRRSV-2 strains, this event involved recombination between lineage 8 and lineage 1. In conclusion, this analysis enhances our understanding of the prevalence and genetic variation of PRRSV-2 in China. These findings provide significant insights for the development of effective prevention and control strategies for PRRS and serve as a foundation for future research in this field.

## 1. Introduction

Porcine reproductive and respiratory syndrome (PRRS) is a highly contagious disease caused by the porcine reproductive and respiratory syndrome virus (PRRSV) (Yu et al., 2021). PRRSV mainly causes spontaneous abortion, stillbirth, and fetal mummification in pregnant sows. Additionally, respiratory insufficiency, interstitial pneumonia, and immunosuppression are also common symptoms in piglets (Li et al., 2022). PRRS was first discovered in the United States in 1987 and rapidly spread worldwide after its detection in Europe in 1990, causing significant economic losses to the global pig industry (Liang et al., 2022). PRRSV was first identified in China in 1996, and since then, it has been reported throughout the country (Guo et al., 2018). In 2006, a highly pathogenic PRRSV (HP-PRRSV) outbreak occurred in China, causing a significant increase in the severity of lesions and mortality rates among infected pigs (Yan et al., 2021).

PRRSV is a single-stranded positive-sense RNA virus that belongs to the order *Nidovirales*, family *Arteriviridae*, genus *Betaarterivirus*, and subgenus *Ampobarterivirus* (King et al., 2018). The genome of PRRSV is approximately 15 kb in length and comprises 11 open reading frames (ORFs) (Fang et al., 2012). ORF1a, ORF1b, and ORF1aTF encode two crucial viral replicase proteins (pp1a and pp1ab), which are further processed into 16 nonstructural proteins (NSPs) under the action of proteases (Sun et al., 2022; Xu et al., 2022). The remaining ORFs (ORF2 ~ 7) encode eight structural proteins, of which GP5 is the most variable, and thus GP5 is often used for genetic variation analysis (Zhao et al., 2022).

PRRSV was previously categorized into two genotypes: PRRSV-1 (represented by the Lelystad virus) and PRRSV-2 (represented by ATCC VR-2332) (Ma et al., 2021; Trevisan et al., 2022). Currently, PRRSV-1 and PRRSV-2 are divided into two distinct species, *Betaarterivirus suid 1* and *Betaarterivirus suid 2* (Brinton et al., 2021). Shi et al. established a classification system for global PRRSV-2 strains by conducting phylogenetic analysis of the ORF5 sequence, PRRSV-2 was divided into nine lineages and 37 sub-lineages. Notably, prevalent strains in China are typically classified into lineages 1, 3, 5, and 8 (Shi et al., 2010; Bao and Li, 2021). Lineage 1 is represented by NADC30, NADC34, JL580, and RFLP 1–4-4 strains. Lineage 3 strains of PRRSV-2 are more commonly found in the southern regions of China. These strains are recognized for their lower level of pathogenicity when compared to other lineages, which is represented by QYYZ and GM2 strains. Lineage 5 (VR-2332-like) has not caused an epidemic in China yet, despite appearing as early as 1996. Lineage 8 is represented by CH-1a, TJ, JXA1, and HUN4 strains, which is further divided into lineages 8.1, 8.7, and 8.9. Lineage 8.1, represented by CH-1a, is also known as Chinese classical PRRSV (C-PRRSV) and was first discovered in China in 1996. While lineage 8.7, represented by TJ, JXA1, and HUN4 strains, is known as the HP-PRRSV (Zhou et al., 2018; Fang et al., 2022; Zhao et al., 2022). PRRSV adapts to changing environmental pressures through mutation and recombination, making disease prevention and control more difficult. *GP5* (*GP5a*) is highly variable and thus is commonly used as a target gene for analyzing genetic variation in PRRSV.

This study intends to reveal the genetic variation and recombination of PRRSV-2 strains in China from 1996 to 2022. A total of 517 PRRSV-2 GP5 sequences were selected, the nucleotide and amino acid homologies of PRRSV-2 *GP5* (*GP5a*) were compared among 60 strains of various lineages and phylogenetic trees were constructed. Furthermore, the recombination events present in the PRRSV-2 GP5 sequences were analyzed, and the evolutionary relationship of PRRSV-2 GP5 was determined in this study to provide a theoretical foundation for the further monitoring of genetic variations of PRRSV in China.

## 2. Materials and methods

### 2.1. The dataset

A total of 517 *GP5* sequences of PRRSV-2 strains were selected (501 PRRSV-2 strains from China and 16 PRRSV-2 strains from the United States were included) from the GenBank database on the NCBI website, including lineages 1, 3, 5, and 8 (Supplementary Table S1). These strains encompassed various years ranging from 1996 to 2022 and comprised the majority of PRRSV strains from China, as well as vaccine strains and representative US strains. Genetic variation and recombination analyses of the *GP5* sequences of PRRSV-2 strains over the past two decades can help to understand the evolution of PRRSV *GP5* in China and provide a theoretical basis for the prevention and control of PRRSV. The *GP5* (*GP5a*) sequences of 60 PRRSV strains selected from 517 PRRSV-2 strains were further analyzed. These strains were carefully selected from lineages 1, 3, 5, and 8, and comprised representative, vaccine, and epidemic strains to ensure a comprehensive analysis of the genetic variation of PRRSV-2 in China (Table 1).

**Table 1:** The GP5 (GP5a) reference sequence of 60 PRRSV strains.

Year	Area	Strain	Genbank accession number
1993	USA	ATCC VR-2332	U87392
1996	China	CH-1a	AY032626
1998	USA	RespPRRS MLV	AF066183
1999	USA	MLV RespPRRS/Repro	AF159149
2000	China	BJ-4	AF331831
2005	USA	MN184A	DQ176019
2005	USA	MN184B	DQ176020
2006	China	R98	DQ355796
2006	China	CC-1	EF153486
2006	China	JXA1	EF112445
2006	China	HUN4	EF635006
2006	China	TJ	EU860248
2006	China	HUB1	EF075945
2006	China	HUB2	EF112446
2006	China	JX143	EU708726
2006	China	S1	DQ459471
2007	USA	MN184C	EF488739
2007	China	DY	JN864948
2007	China	SHH	EU106888
2007	China	GD	EU109503
2007	China	Henan-1	EU200962
2007	China	BJ	EU825723
2007	China	XH-GD	EU624117
2008	USA	NADC30	JN654459
2008	USA	NADC31	JN660150
2008	China	PRRSV01	FJ175687
2008	China	PRRSV02	FJ175688
2008	China	PRRSV03	FJ175689
2008	China	CH-1R	EU807840
2008	China	JXA1 P80	FJ548853
2008	China	WUH3	HM853673
2009	China	GS2002	EU880441
2009	China	GS2003	EU880442
2009	China	GS2004	EU880443
2009	China	CH2002	EU880438
2009	China	CH2003	EU880440
2009	China	CH2004	EU880439
2009	China	JXA1-P120	KC422727
2009	China	ZP-1	HM016159
2009	China	SX-1	GQ857656
2009	China	SD1-100	GQ914997
2010	China	QY2010	JQ743666
2010	China	JX	JX317649
2011	China	GM2	JN662424
2011	China	QYYZ	JQ308798
2011	China	YN-2011	JX857698
2011	China	WUH4	JQ326271
2013	China	JL580	KR706343
2014	China	CHsx1401	KP861625
2014	China	FJ1402	KX169191
2014	China	FJM4	KY412888
2015	China	HNyc15	KT945018
2015	China	HNjz15	KT945017
2015	China	SD-A19	MF375260
2015	China	FJFS	KP998476
2015	China	GDsg	KX621003
2017	China	SD17-36	MH121061
2017	China	SD17-38	MH068878
2020	China	rJXA1-R	MT163314
2021	China	ZJqz21	OK274266

### 2.2. Sequence analysis of PRRSV *GP5* (*GP5a*) gene

The Clustal W method in the MegAlign function of DNAStar software (version 7.0, Madison, WI) was used to analyze the nucleotide homology of the *GP5 (GP5a)* gene. The *GP5* (*GP5a*) nucleotide sequences were translated into the corresponding amino acid sequences and then analyzed for amino acid homology of the *GP5* (*GP5a*) gene using the Clustal W method in the MegAlign function of DNAStar software. In addition, amino acid sequences were compared using MegAlign.

### 2.3. Phylogenetic analysis

Phylogenetic analysis of the *GP5* gene was based on the reference strains information shown in Table S1. The phylogenetic trees were constructed in MEGA software (version 7.0.26, Mega Limited, Auckland, New Zealand) using the neighbor-joining (NJ) and maximum-likelihood (ML) methods, with 1,000 bootstrap replicates performed by the NJ method and 100 bootstrap replicates by the ML method, and the remaining parameters were set to default values. The generated phylogenetic trees were then embellished and annotated using Interactive Tree of Life.^1^

### 2.4. Recombination analysis

In this study, we utilized RDP software (version 4.0) to analyze potential recombination events, employing a comprehensive set of seven computational methods including RDP, GENECONV, BootScan, MaxChi, Chimera, SiScan, and 3 eq to identify potential recombinant events. Those strains that met the criteria of being identified as genetic recombination by four or more of these methods and the reliability of the *p*-value is less than 0.05 were considered as recombinant strains. Furthermore, the detected recombinant events were further verified using SimPlot software (version 3.5.1).

## 3. Results

### 3.1. Nucleotide and amino acid homologies of the *GP5* gene

In order to further understand the genetic variation of the PRRSV *GP5* gene, 60 PRRSV strains were selected from lineages 1, 3, 5, and 8, respectively, and nucleotide and amino acid homologies analysis of the PRRSV *GP5* were performed by DNAStar software to determine the evolutionary relationship between different lineages. The study revealed that the nucleotide homologies of the *GP5* gene in 60 PRRSV strains ranged from 81.4 to 100.0%, and the amino acid homologies of the *GP5* gene ranged from 78.1 to 100.0%. The CHsx1401-2014 strain and HNyc15-2015 strain, NADC31-2008 strain and GDsg-2015 strain exhibited the lowest nucleotide homology of 81.4%, the HUN4-2007 strain showed the lowest amino acid homology of 78.1% with the GS2002-2009 and GS2003-2009 strains. The ZJqz21-2021 strain and NADC30-2008 strain, QYYZ-2011 strain and QY2010-2010 strain, and RespPRRS MLV-1998 strain and MLV RespPRRS-Repro-1999 strain had the highest nucleotide and amino acid homologies of 100.0% (Supplementary Figures S1, S2).

In addition, nucleotide and amino acid homologies analysis of the PRRSV GP5 sequences in the four lineages was performed respectively, the results showed that the largest difference in nucleotide and amino acid homologies were found among the lineage 1 strains (81.4 ~ 100.0%, 80.6 ~ 100.0%), and the smallest difference in nucleotide and amino acid homologies were found among the lineage 5 strains (98.3 ~ 100.0%, 96.0 ~ 100.0%) (Table 2).

**Table 2:** Nucleotide and amino acid homologies analysis based on *GP5* gene (%).

		Lineage 1	Lineage 3	Lineage 5	Lineage 8
Lineage 1	nt	**81.4**^**a**^ ~ **100.0**^**b**^	81.4 ~ 89.6	82.3 ~ 87.1	82.9 ~ 87.7
	aa	80.6 ~ **100.0**^**b**^	81.0 ~ 90.0	**78.1**^**a**^ ~ 86.1	80.6 ~ **100.0**^**b**^
Lineage 3	nt		96.2 ~ **100.0**^**b**^	82.9 ~ 84.4	82.8 ~ 85.7
	aa		94.0 ~ **100.0**^**b**^	79.6 ~ 82.6	80.1 ~ 90.0
Lineage 5	nt			98.3 ~ **100.0**^**b**^	87.2 ~ 92.0
	aa			96.0 ~ **100.0**^**b**^	78.1 ~ 91.5
Lineage 8	nt				93.2 ~ **100.0**^**b**^
	aa				82.1 ~ **100.0**^**b**^

^a^Indicates the lowest homology.

^b^Indicates the highest homology.

### 3.2. Nucleotide and amino acid homologies of the *GP5a* gene

The nucleotide and amino acid homologies of the *GP5a* gene from 60 PRRSV strains were analyzed using DNAStar software. The results revealed that the nucleotide homologies of the *GP5a* gene ranged from 78.0 to 100.0%, and the amino acid homologies ranged from 70.2 to 100.0%. The lowest nucleotide and amino acid homologies of *GP5a* were found among the JXA1 P80-2008 strain and the GM2-2011, QY2020-2010, and QYYZ-2011 strains (78.0, 70.2%), while the highest nucleotide homology of 100.0% was found among the WUH3-2008 strain and the JXA1-2006 strain, the ZJqz21-2021 strain and the NADC30-2008, the QYYZ-2011 strain and the QY2010-2010 strain, and the RespPRRS MLV-1998 strain and the MLV RespPRRS-Repro-1999 strain (Supplementary Figures S3, S4). The largest difference in nucleotide and amino acid homologies were found among the lineage 1 strains (79.4 ~ 100.0%, 74.5 ~ 100.0%), the smallest difference in nucleotide homologies were found among the lineage 5 strains (97.2 ~ 100.0%), and the smallest difference in amino acid homologies were found among the lineage 3 strains (95.7 ~ 100.0%) (Table 3).

**Table 3:** Nucleotide and amino acid homologies analysis based on *GP5a* gene (%).

		Lineage 1	Lineage 3	Lineage 5	Lineage 8
Lineage 1	nt	79.4 ~ **100.0**^**b**^	82.3 ~ 90.8	79.4 ~ 90.1	78.7 ~ 92.2
	aa	74.5 ~ **100.0**^**b**^	78.7 ~ 89.4	76.6 ~ 83.0	74.5 ~ 91.5
Lineage 3	nt		95.0 ~ **100.0**^**b**^	80.9 ~ 83.7	**78.0**^**a**^ ~ 86.5
	aa		95.7 ~ **100.0**^**b**^	76.6 ~ 83.0	**70.2**^**a**^ ~ 83.0
Lineage 5	nt			97.2 ~ **100.0**^**b**^	84.4 ~ 91.5
	aa			93.6 ~ **100.0**^**b**^	72.3 ~ 85.1
Lineage 8	nt				87.9 ~ **100.0**^**b**^
	aa				83.0 ~ **100.0**^**b**^

^a^Indicates the lowest homology.

^b^Indicates the highest homology.

### 3.3. *GP5* (*GP5a*) amino acid sequence alignment

**Figure 1:**
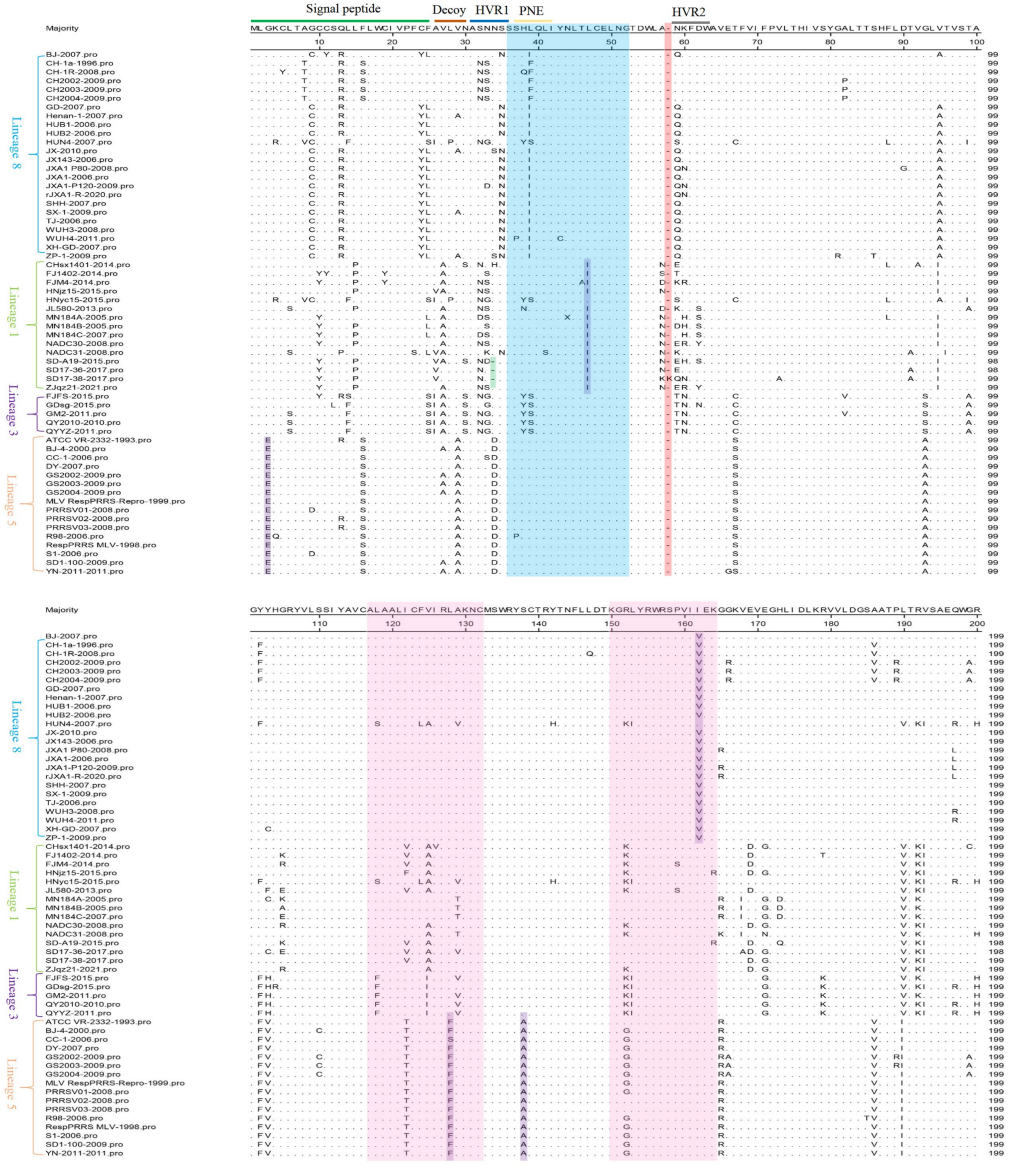
60 PRRSV *GP5* amino acid sequences alignment in lineages 1, 3, 5, and 8. The blue region represents the neutralizing epitope, the pink region represents the two T-cell antigenic regions, the purple region represents lineage-specific mutations, the green region represents deleted amino acids, and the red region represents inserted amino acids.

The MegAlign function of DNAStar software was used to analyze the amino acid mutation sites of 60 PRRSV *GP5* (*GP5a*) gene in this study. The results indicated that the amino acid sites of the GP5 were prone to mutation, most of these mutations were observed in the signal peptide region, two highly variable regions (HVRs), and near two T-cell antigenic sites. The N-glycosylation sites located at the 32nd, 33rd, 34th, and 35th positions of HVR1 were found to be mutated, with lineage 5 exhibiting an S^34^ → D^34^ mutation and lineage 8 exhibiting an S^35^ → N^35^ mutation. Furthermore, the results also showed that three strains (SD-A19-2015, SD17-36-2017, and SD17-38-2017) had a deletion at the 34^th^ position, and the SD17-38-2017 strain had an amino acid insertion at the 58^th^ position. 27 strains showed a Q^13^ → R^13^ mutation, while one strain showed a Q^13^ → P^13^ mutation. However, no mutation was observed at the 151^st^ amino acid position. 45 strains had mutations in the neutral epitope region, including mutations in lineages 1, 3, and 8, and one mutation in lineage 5. Some mutations at amino acid positions were specific to a particular lineage, such as L^47^ → I^47^ and I^162^ → V^162^ mutations in lineage 8, and G^3^ → E^3^, L^128^ → F^128^, and S^138^ → A^138^ mutations in lineage 5 (Figure 1).

**Figure 2:**
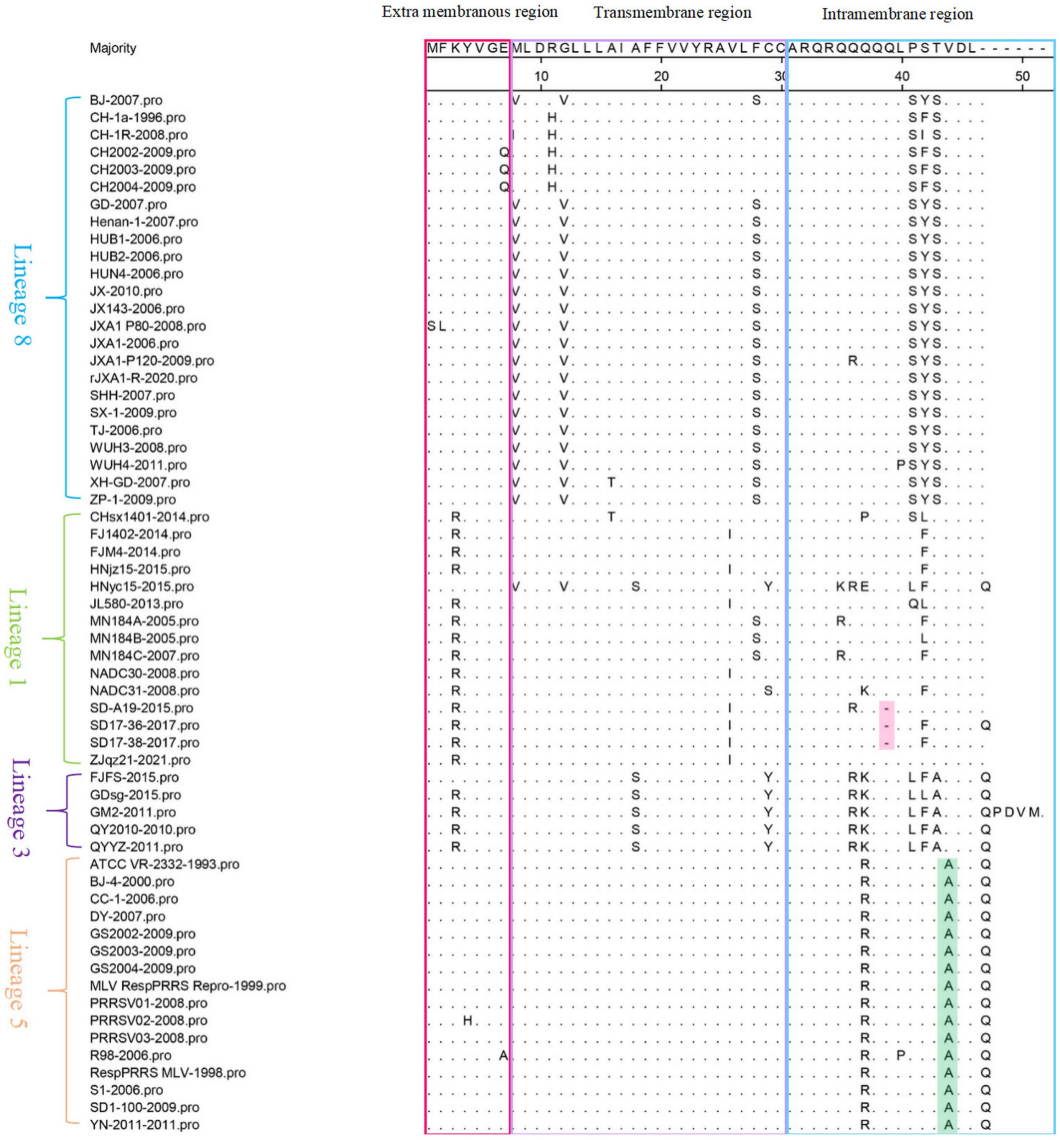
60 PRRSV GP5a amino acid sequences alignment in lineages 1, 3, 5, and 8. The green region represents lineage-specific mutations, the purple region represents deleted amino acids.

GP5a was mutated mainly at the 3rd, 8th, 12th, 28th, 37th, 41st, 42nd, and 43rd amino acid positions. The K^3^ → R^3^ mutation occurred in the extra membranous region, the M^8^ → V^8^/I^8^, G^12^ → V^12^, and F^28^ → S^28^ mutations occurred in the transmembrane region, and the Q^37^ → P^37^/E^37^/K^37^/R^37^, P^41^ → S^41^/L^41^/Q^41^, S^42^ → Y^42^/F^42^/I^42^/L^42^, and T^43^ → S^43^ mutations occurred in the intramembrane region. This indicates that the mutations in GP5a are mainly concentrated in the transmembrane and intramembrane regions. In addition, all the lineage 5 strains showed a V^44^ → A^44^ mutation, which is lineage-specific. SD-A19-2015, SD17-36-2017, and SD17-38-2017 strains had a deletion at the 39^th^ amino acid position (Figure 2).

### 3.4. Phylogenetic analysis

**Figure 3:**
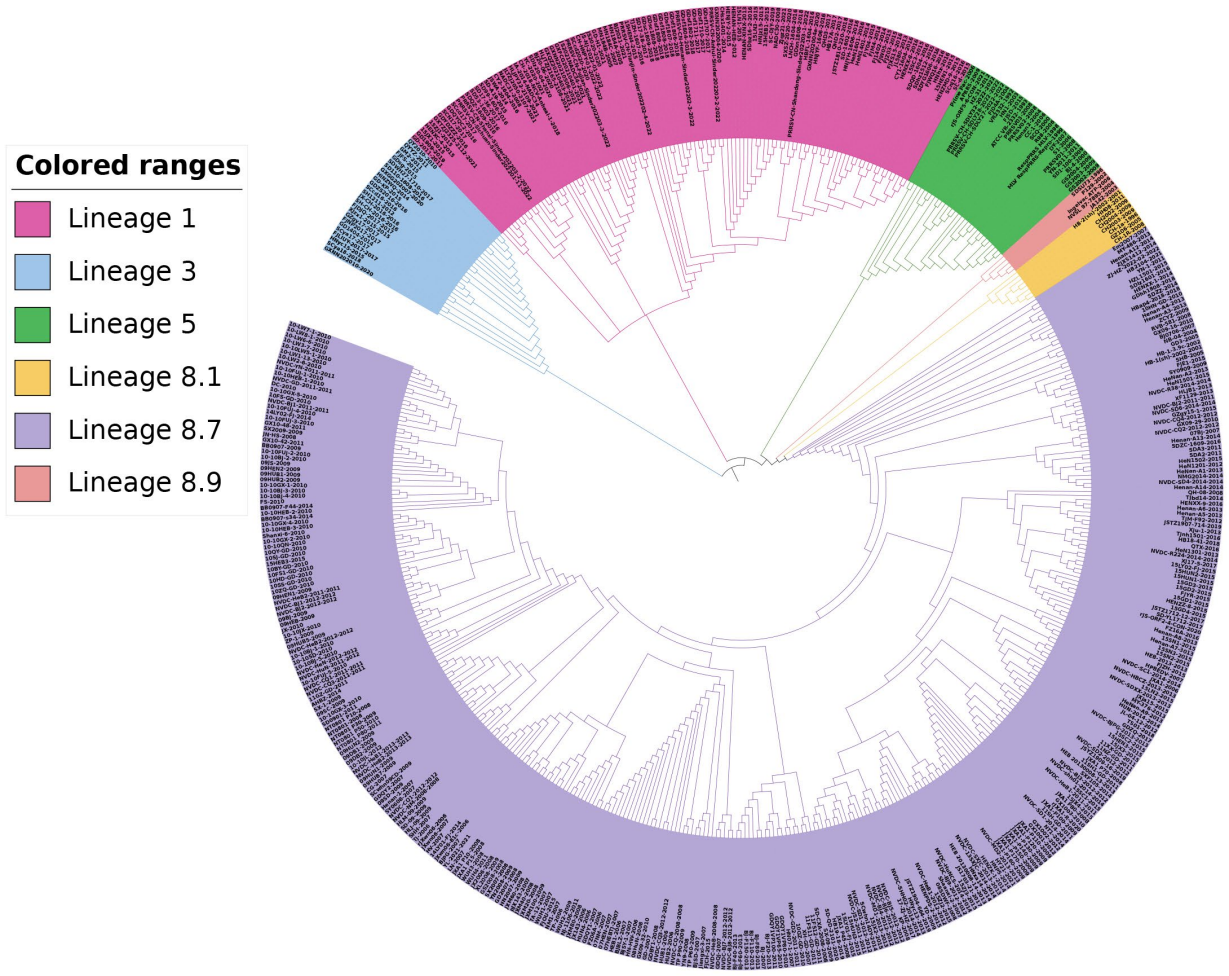
Phylogenetic analysis based on the *GP5* gene. The NJ method was used to construct the phylogenetic tree using MEGA software (version 7.0.26, Mega Limited, Auckland, New Zealand) with 1,000 bootstrap replicates.

**Figure 4:**
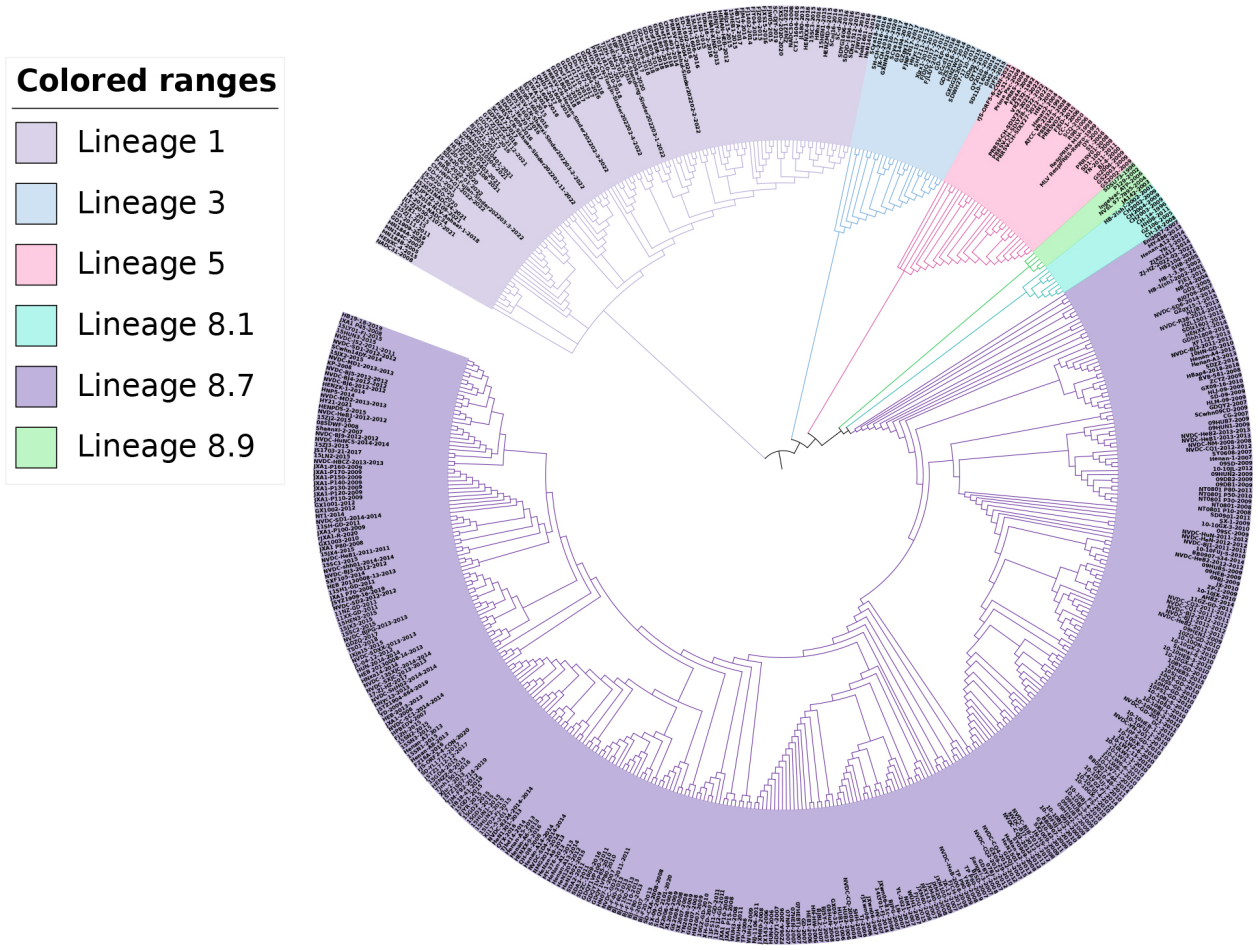
Phylogenetic analysis based on the *GP5* gene. The ML method was used to construct the phylogenetic tree using MEGA software (version 7.0.26, Mega Limited, Auckland, New Zealand) with 100 bootstrap replicates.

For the phylogenetic analysis, a total of 517 PRRSV-2 strains were selected based on the global PRRSV classification system, and the GP5 sequence information was retrieved from the GenBank database. The results of the phylogenetic analysis revealed that the PRRSV-2 strains prevalent in China could be classified into lineages 1, 3, 5, and 8. Among these, lineage 1 and lineage 8 were the current main prevalent lineages. Lineage 5 and lineage 8 were genetically close. Additionally, the PRRSV-2 strains newly added in 2022 belonged to lineage 1 and lineage 5 in the phylogenetic trees (Figures 3, 4).

### 3.5. Recombinant analysis

**Figure 5:**
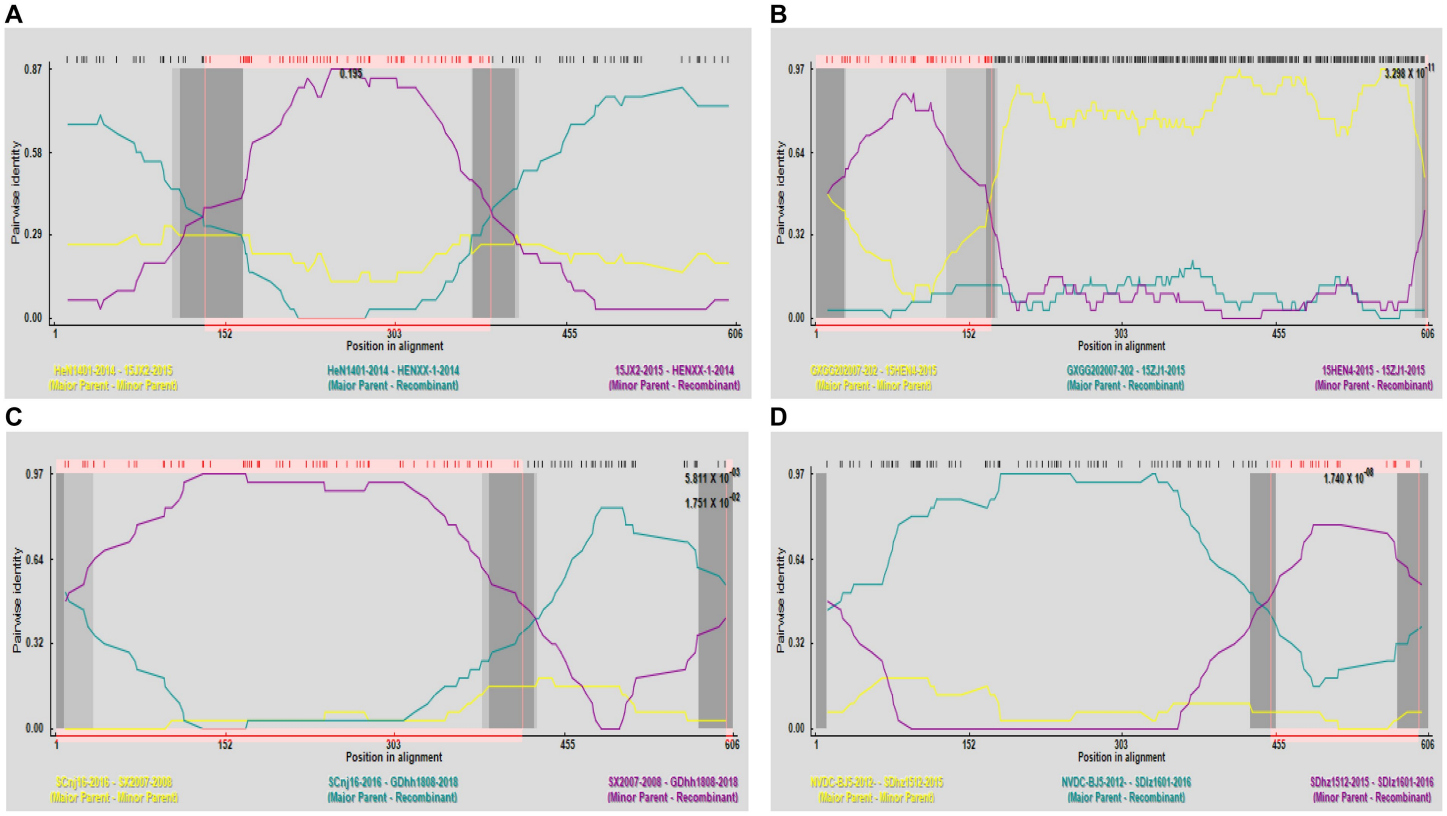
Prediction of *GP5* gene recombination events by RDP. **(A)** Recombination analysis results of the recombinant strain HENXX-1-2014. **(B)** Recombination analysis results of the recombinant strain 15ZJ1-2015. **(C)** Recombination analysis results of the recombinant strain GDhh1808-2018. **(D)** Recombination analysis results of the recombinant strain SDlz1601-2016.

**Figure 6:**
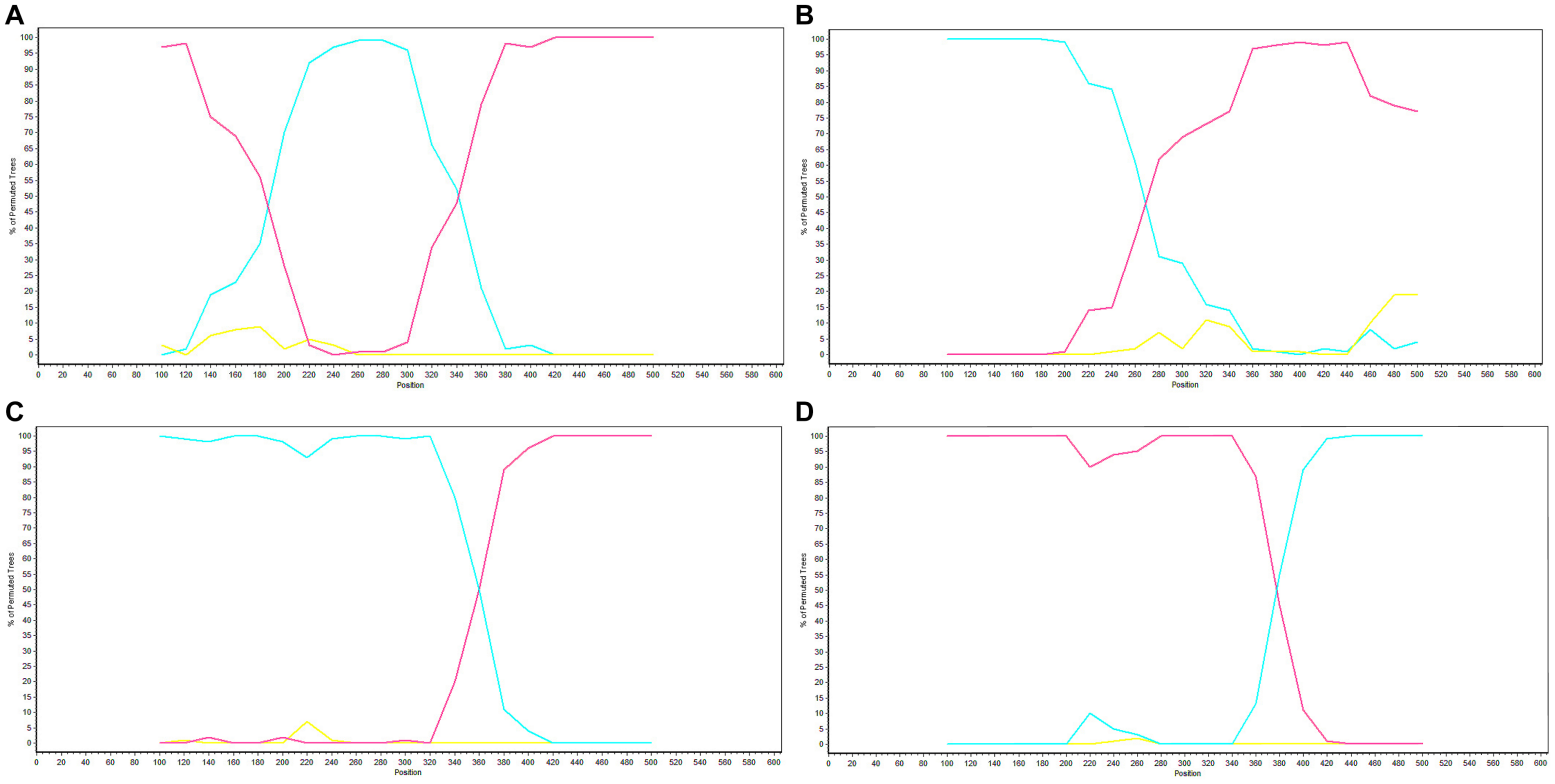
Validation of *GP5* gene recombination events by SimPlot. **(A)** Recombination validation results of the recombinant strain HENXX-1-2014. **(B)** Recombination validation results of the recombinant strain 15ZJ1-2015. **(C)** Recombination validation results of the recombinant strain GDhh1808-2018. **(D)** Recombination validation results of the recombinant strain SDlz1601-2016. The red line represents the major parent strain, the blue line represents the minor parent strain, and the yellow line represents the control strain (CH-1a).

Potential recombination events were identified using RDP software and validated by SimPlot. The analysis of *GP5* gene recombination detected four potential recombination events using RDP software, among which, one recombination event was validated by SimPlot. The RDP results indicated that the four recombinant events had high reliability, with a *p*-value of less than 0.05, indicating statistical significance. Events 1, 3, and 4 were identified in lineage 8 strains, indicating the recombination of lineage 8 and lineage 1. Recombinant event 2 was identified in lineage 1 strains, indicating recombination of lineage 3 and lineage 1 (Table 4). The recombinant strain HENXX-1-2014 had HeN1401-2014 and 15JX2-2015 as the major and minor parent strains, respectively (Figure 5). RDP detected 15ZJ1-2015 as a recombinant strain between lineage 3 and lineage 1, and GDhh1808-2018 and SDlz1601-2016 as recombinant strains between lineage 8 and lineage 1. However, SimPlot did not validate the existence of these three recombination events (Figure 6).

**Table 4:** Recombination analysis of *GP5* gene.

Recombination event	Recombinant strain (lineage)	Main parental strain (lineage)	Minor parental strain (lineage)	Recombinant breakpoint	Recombination analysis method
1	HENXX-1-2014 (8)	HeN1401-2014 (1)	15JX2-2015 (8)	105–168 (371–413)	RDP (*P*=NS)
					GENECONV (*P*=NS)
					BootScan (*P*=NS)
					MaxChi (*p* = 2.274*10^−5^)
					Chimaera (*p* = 1.546*10^−4^)
					SiScan (*p* = 1.176*10^−3^)
					3seq (*p* = 2.032*10^−12^)
2	15ZJ1-2015 (1)	GXGG202007-2020 (3)	15HEN4-2015 (1)	31–593 (130–180)	RDP (*p* = 1.461*10^−5^)
					GENECONV (*p* = 7.208*10^−5^)
					BootScan (*p* = 2.670*10^−7^)
					MaxChi (*p* = 1.336*10^−2^)
					Chimaera (*p* = 2.339*10^−2^)
					SiScan (*p* = 7.914*10^−33^)
					3seq (*p* = 2.617*10^−8^)
3	GDhh1808-2018 (8)	SCnj16-2016 (1)	SX2007-2008 (8)	34–575 (382–431)	RDP (*p* = 1.751*10^−2^)
					GENECONV (*P*=NS)
					BootScan (*P*=NS)
					MaxChi (*p* = 1.257*10^−4^)
					Chimaera (*P*=NS)
					SiScan (*p* = 4.762*10^−12^)
					3seq (*p* = 7.626*10^−9^)
4	SDlz1601-2016 (8)	NVDC-BJ5-2012 (8)	SDlz1512-2015 (1)	430–455 (11–575)	RDP (*p* = 1.740*10^−8^)
					GENECONV (*p* = 6.233*10^−7^)
					BootScan (*p* = 1.421*10^−8^)
					MaxChi (*p* = 8.311*10^−3^)
					Chimaera (*p* = 7.480*10^−3^)
					SiScan (*p* = 2.225*10^−5^)
					3seq (*p* = 1.499*10^−7^)

NS, no significant *p*-value.

## 4. Discussion

In recent years, the high rate of mutation and frequent recombination of PRRSV have exacerbated the epidemic of PRRS and increased the difficulty of disease prevention and control. The *GP5* (*GP5a*) is highly variable and plays an extremely important role in the process of genetic evolution. Therefore, GP5 (GP5a) has become an essential indicator for identifying and analyzing PRRSV.

In order to understand the genetic variability of PRRSV *GP5* (*GP5a*) and establish the evolutionary relationship among different lineages, this study involved the selection of 60 PRRSV strains for nucleotide and amino acid homologies analysis, as well as amino acid sequence alignment. The analysis of nucleotide and amino acid homologies of PRRSV-2 *GP5* (*GP5a*) revealed that the nucleotide homologies range of 81.4 ~ 100.0% (78.0 ~ 100.0%) and the amino acid homologies range of 78.1 ~ 100.0% (70.2 ~ 100.0%) among the 60 PRRSV-2 strains. Among these, lineage 1 has the largest nucleotide homology difference, indicating that there may have been extensive recombination and mutation between these strains. Conversely, lineage 3 and lineage 5 have relatively small nucleotide homology differences. Lineage 3 emerged relatively late in China and has a lower clinical detection rate compared to other lineages. Lineage 5 has been prevalent in China for a considerable period, but its representative strains such as ATCC VR2332 and BJ-4 are not dominant in the field, and their pathogenicity is relatively weak, resulting in a low clinical detection rate (Guo et al., 2018). It is speculated that these strains have a low probability of mutation during the evolutionary process. Lineage 8 strains are the predominant circulating strains of PRRSV in China. One of their unique features is their high recombination frequency, which enables them to adapt to constantly changing environmental pressures over extended periods of evolution. Lineage 1 strains have the largest amino acid homology difference, whereas lineage 3 and lineage 5 strains have relatively small amino acid homology differences. This is consistent with the results of nucleotide homology, so we speculate that the *GP5* (*GP5a*) protein has undergone genetic mutations accordingly.

Substitutions, deletions, and insertions are crucial factors in the evolution of PRRSV. The *NSP2* and *GP5* (*GP5a*) genes are the most variable genes in the PRRSV genome and are therefore the primary focus of studies on PRRSV genetic variation. The *NSP2* gene is highly variable among the NSPs. GP5 protein has a wide range of variations in the signal peptide region, inducible epitope, neutralizing epitope, and HVRs (Luo et al., 2023). As the major structural protein with the highest degree of variation in PRRSV, *GP5* has become one of the indicators for monitoring PRRSV variation (Deng et al., 2016). In the amino acid sequence alignment analysis, mutations in several amino acid sites in the GP5 protein of PRRSV-2 strains were identified in this study. These mutations were primarily found in the signal peptide region, two HVRs, and the vicinity of two T-cell antigenic sites. A total of 45 strains had mutations in the neutralization epitope region, including strains from lineages 1, 3, and 8, as well as one strain had a mutation in lineage 5. Mutations in the neutralization epitope region of the virus can result in immune evasion, thus reducing the protective efficacy of the vaccine (Li et al., 2022). Glycosylation of the GP5 is the primary mechanism of virus immune escape and persistent infection, leading to a decrease in the body’s immunity and antibody production ability (Ansari et al., 2006). Our study showed that mutations in the N-glycosylation sites located at the 32nd, 33rd, 34th, and 35th positions of HVR1. Furthermore, the study identified specific amino acids at the 13th position of the GP5 protein, serving as a key site associated with virulence-related traits. Our analysis exhibited Q^13^ → R^13^ and Q^13^ → P^13^ mutations, but no mutation was found at the 151st amino acid position, mutations at the 13th amino acid position may change the virulence of the virus and increase its ability to evade immunity (Wesley et al., 1998). Deletion is considered a characteristic of new strains, three strains (SD-A19-2015, SD17-36-2017, and SD17-38-2017) had a deletion at the 34th amino acid position. Fan et al. (Fan, 2017) also discovered that the PRRSV virulent strains SD7, SD8, and SD9 had a deletion at the 34th amino acid position. In addition, we observed the SD17-38-2017 strain exhibited an insertion at the 58th amino acid position, and studies are required to further investigate the effects of the insertion on these strains. The *GP5a* protein is critical for viral viability and infectivity, but studies on genetic variation in the GP5a protein are scarce. In this study, we analyzed mutations in *GP5a*, which encodes 47–52 amino acids, and the mutation sites are mainly concentrated in the transmembrane and intramembrane regions. The mutation is the main driving force of PRRSV evolution, which can result in the immune escape of the virus and thus cause vaccine immunization failure. This poses a significant challenge for future PRRS vaccine development. Monitoring the mutations in the *GP5* (*GP5a*) protein is crucial to understanding the future development trends of PRRSV and providing a foundation for vaccine development.

Phylogenetic analysis revealed that the prevalent PRRSV-2 strains in China were divided into lineages 1, 3, 5, and 8. Lineage 1 and lineage 8 dominated the epidemic of PRRS, lineage 5 and lineage 8 had a close genetic relationship, while lineage 3 and lineage 8 were genetically distant in the NJ phylogenetic tree, lineage 1 and lineage 8 were genetically distant in the ML phylogenetic tree. Our study compared the genetic variation of the *GP5* gene with the highly variable *NSP2* gene and the conserved *NSP4* gene (Sha et al., 2023; Zhang et al., 2023). In this study, lineage 1 and lineage 8 dominated the epidemic of PRRS, which is consistent with the results of these two studies mentioned above. Lineage 5 was genetically closer to lineage 8, while lineage 3 was genetically further apart from lineage 8 in the NJ phylogenetic tree, and lineage 1 and lineage 8 were genetically distant in the ML phylogenetic tree. These differences in genetic distance may be related to the strain numbers, different genes, and methods chosen for analysis. Yin et al. (2021) employed the NJ method to construct a phylogenetic tree using the *ORF5* gene. The results showed that lineage 3 and lineage 1 were closely related and located in the same branch, while lineage 5 and lineage 8 were genetically distant. It is speculated that the differences in the phylogenetic trees may be related to the number, year, and range of selected strains. The newly added PRRSV-2 strains in 2022 belonged to lineage 1 and lineage 5, indicating that the prevalence of lineage 1 strains is becoming more widespread. Currently, the prevalent NADC30-like and NADC34-like strains belong to lineage 1, while lineage 5 strains had a low clinical detection rate in the past. Based on the results of this study, it can be inferred that the detection rate of lineage 5 strains is likely to increase gradually in the future.

Since the first emergence of PRRSV in China in 1996, it has been rapidly evolving and widely spreading throughout the country. The PRRSV NADC30 strain was initially detected in the United States in 2008. Subsequently, in 2015, PRRSV NADC30-like strains had become widespread epidemic strains. Recombination among NADC30, C-PRRSV, and HP-PRRSV strains has resulted in different PRRSV NADC30-like strains exhibiting different pathogenicity (Dong et al., 2018; Wang et al., 2018). Recombination in PRRSV not only leads to the emergence of new strains but is also linked to increased virulence (Yu et al., 2020). Chen et al. (2021) discovered a new natural recombinant strain, HBap4-2018, with HP-PRRSV and NADC30-like serving as the main and minor parent strains, respectively. The mortality rate among piglets inoculated with HBap4-2018 was as high as 60%, which suggests that the strain is highly pathogenic to piglets. Similarly, Xia et al. (2023) isolated a new HP-PRRSV strain (SD2020) from sick pigs suspected of HP-PRRS in a pig farm. They demonstrated that SD2020 was a natural recombinant strain of the vaccine strain VR-2332 and JXA1-like virus strain. The two splice fragments in the viral genome that are highly homologous to JXA1 may have originated from the wild strain JXA1 and the vaccine strain JXA1-R, respectively. The recombination event of SD2020 and its mutation site may be associated with its high pathogenicity. It is essential to note that natural recombination events can also take place between the vaccine strains and wild strains, and this should be given utmost importance. Lu et al. (2012) demonstrated that GM2 was identified as a potential recombinant between the vaccine strain MLV RespPRRS/Repro and the emerging prototype wild strain QYYZ in China. Similarly, Wang et al. (2019) found that IA70388-R was a natural recombinant strain from the PRRSV vaccine strain Fostera and wild strain IA76950-WT. The occurrence of these recombination events suggests that recombination is critical in the evolution of PRRSV.

The recombination analysis in this study indicated that there was one recombination event, and most of the recombinant strains identified by RDP exhibited recombination between lineage 8 and lineage 1. Yin et al. (2021) conducted a recombination analysis of the *ORF5* gene and identified four recombinant strains that belong to lineage 1. The analysis revealed that the major parental strain belonged to lineage 1, while the minor parental strain belonged to lineage 8, indicating that recombination between lineage 8 and lineage 1 strains was prevalent. In addition, Li et al. (2022) also conducted a recombination analysis based on the *ORF5* gene, and RDP identified four recombination events, with both the major and minor parental strains belonging to lineage 1. Recombination is a significant driving force in the evolution of PRRSV, allowing the virus to adapt to environmental stresses. However, it can also reduce the efficacy of vaccines, making PRRS prevention and control more challenging.

PRRS is a highly prevalent disease in China with a wide variety of strains. The epidemic of PRRS in China can be classified into three distinct phases, each characterized by different strains and clinical manifestations. From 1996 to 2006, C-PRRSV strains were the most prevalent strains, and these strains primarily caused clinical abortion in gestating sows and respiratory symptoms in piglets. From 2006 to 2012, a new and more virulent strain called HP-PRRSV emerged as the predominant strain. It resulted in more severe clinical symptoms and affected not only piglets but also fattening pigs and adult pigs. The disease caused by HP-PRRSV had a high morbidity and mortality rate, leading to significant economic losses to the pig industry in China. From 2012 to the present, there has been a noticeable increase in the diversity of PRRSV strains in swine herds. An important trend is the rapid increase in the proportion of lineage 1 strains, which have become the dominant strain. Since 2012, NADC30-like strains have become prevalent in China. The clinical symptoms of these strains are highly variable, making it difficult to diagnose and control. Furthermore, existing commercially available modified live vaccines do not provide adequate protection (Yu et al., 2021). The emergence of recombinant strains has further complicated the prevention and control of PRRS, posing a great challenge to the Chinese pig industry. In 2017, the NADC34-like strains emerged as moderately virulent and widespread strains with epidemic potential, causing severe reproductive disorders in sows. Since 2020, the detection rate of NADC34-like strains has significantly increased (Zhao et al., 2022). Currently, the predominant strains in China are mainly NADC30-like and NADC34-like strains of lineage 1, followed by HP-PRRSV strains. In 2020, new strains called PRRSV 1–4-4 L1C were discovered in the United States. These strains are more transmissible and have a higher mortality rate in sows and piglets than previous strains. It also exhibited characteristic lesions of thymus and lymph node necrosis (Trevisan et al., 2021).

In this study, we analyzed the genetic variation and recombination of the PRRSV-2 *GP5 (GP5a)* gene in China from 1996 to 2022. We performed sequence analysis of *GP5* (*GP5a*), clarified the evolutionary relationship between the different lineages at the nucleotide and amino acid levels, and hypothesized that the detection rate of the lineage 5 strains would gradually increase in the future, and the occurrence of the GP5 recombination events would be more common. PRRSV mutants can adapt to environmental changes, resulting in new survival strategies. Existing vaccines offer limited protection against the disease, which makes the control of PRRS even more challenging. There is an urgent need to develop new vaccines or drugs that are resistant to these mutant strains. In the future, the occurrence of mutations and recombinations of PRRSV-2 *GP5* (*GP5a*) is expected to become more common. Therefore, effective prevention and control of the disease will continue to pose a major challenge.

## 5. Conclusion

From 1996 to 2022, PRRSV-2 strains circulating in China have been classified into lineages 1, 3, 5, and 8. Among these, lineage 8 and lineage 1 are the most widespread and susceptible to recombination. The *GP5* (*GP5a*) amino acid mutation is a major driver of PRRSV evolution and contributes to viral evolution. Therefore, in order to enhance disease prevention and control measures in the future, it is imperative to intensify monitoring of PRRSV genetic variations.

## Data availability statement

The datasets presented in this study can be found in online repositories. The names of the repository or repositories and accession number(s) can be found in the article or Supplementary material.

## Author contributions

QL, YZ, and YH collected the data and wrote the original draft. HZ, GL, HS, and ZZ reviewed and revised the draft. LH and MZ made the final revision of the manuscript. All authors contributed to the article and approved the submitted version.

## Funding

This research was funded by the National Natural Science Foundation of China (31902279).

## Conflict of interest

The authors declare that the research was conducted in the absence of any commercial or financial relationships that could be construed as a potential conflict of interest.

## Publisher’s note

All claims expressed in this article are solely those of the authors and do not necessarily represent those of their affiliated organizations, or those of the publisher, the editors and the reviewers. Any product that may be evaluated in this article, or claim that may be made by its manufacturer, is not guaranteed or endorsed by the publisher.

## Footnotes

^1^https://itol.embl.de
